# Control Beliefs & Awareness of Age Related Change in Middle-Aged and Older Adults With Cancer

**DOI:** 10.1093/geroni/igaf122.1211

**Published:** 2025-12-31

**Authors:** Kathryn Swaim, Shevaun Neupert

**Affiliations:** North Carolina State University, Raleigh, North Carolina, United States; North Carolina State University, Raleigh, North Carolina, United States

## Abstract

Control beliefs have been defined as the belief that one can achieve their goals. As people age, the likelihood of experiencing physical changes, and serious illness increases which has been found to impact control over time. While aging and illness have been seen as normative parts of aging, how people perceive these changes may impact health and well-being overtime. Awareness of age related change (AARC), highlights not only losses (e.g., physical limitations, illness) but gains (e.g., greater social connections, knowledge and experience, engagement in hobbies) that come with aging. Experiencing daily AARC gains may impact how adults with cancer may feel in control day-to-day. The objective of this study was to investigate moderating variables such as daily uplifts (i.e., any activity or connection that brings joy) in the potential relationship with daily AARC gains and control beliefs in adults with cancer. A sample of 440 U.S. adults aged 50-85 participated in a 14-day daily diary study with 48 reported having been diagnosed with cancer. It was found that on days when adults with cancer reported more AARC gains, they reported feeling more in control (t = 3.29, p = 0.0017). It was also found that on days people reported having few AARC gains and but engaged in more uplifts, they reported feeling more control (t = -2.47, p = 0.0167). Therefore, not only focusing on gains associated with aging, but engaging in uplifting activities can be substantially beneficial for adults navigating cancer and aging.

